# Volumetric Breast Ultrasound as a Screening Modality in Mammographically Dense Breasts

**DOI:** 10.5402/2013/235270

**Published:** 2012-10-23

**Authors:** Vincenzo Giuliano, Concetta Giuliano

**Affiliations:** Breast Cancer Research Institute, Nova Southeastern University College of Osteopathic Medicine, 5732 Canton Cove, Winter Springs, FL 32708, USA

## Abstract

This investigation is part of an ongoing large scale study using volumetric breast ultrasound (VBUS) as a screening modality in mammographically dense breasts, offering a substantial benefit to MR imaging of the breast in terms of cost and efficiency. The addition of VBUS to mammography in women with greater than 50% breast density resulted in the detection of 12.3 per 1,000 breast cancers, compared to 4.6 per 1,000 by mammography alone with an overall attributable risk of breast cancer of 19.92 (95% confidence level, 16.75–23.61) in our screened population. These preliminary results may justify the cost benefit of implementing the judicious use of VBUS as an alternative to MR imaging of the breast in conjunction with mammography in the dense breast screening population.

## 1. Introduction

Mammographic density as an independent risk factor for developing breast cancer has been documented since the 1970s [1]. The appearance of breast tissue is variable among women. The appearance of density on mammography is the result of the relative proportion of breast stroma, which is less radiolucent compared to fat, accounting for increased breast density. Wolfe classified breast density as an independent risk factor for breast cancer in women [2, 3]. Approximately 70 to 80% of breast cancers occur in women with no major predictors [4–6]. Population-based screening for early detection of breast cancer is therefore the primary strategy for reducing breast cancer mortality.

Mammography has been used as the standard imaging method for breast cancer screening, with reduction in breast cancer mortality [7]. Computer-aided detection (CAD) technology with full-field digital mammography (FFDM) has been shown to have several advantages over screen-film mammography, including higher contrast resolution, better dynamic range, and lower noise [8, 9]. Previous studies have shown that CAD performance is similar for the detection of cancer in fatty breasts and dense breasts with screen-film mammography (90% versus 88%, resp.; *P* = 0.38) [10] and with FFDM (95% versus 98%; *P* = 0.537) [11]. Sensitivity in extremely dense breasts was only 60% [12]. There are numerous studies showing that CAD performance is limited by background parenchymal breast density, where the sensitivity of the detection of breast masses sensitivity is significantly higher for fatty breasts than for dense breasts [11, 13–15]. Breast density significantly reduces the ability to visualize cancers on mammography. All false-negative lesions detected with CAD manifested as masses [16]. The number of missed cancers is substantially increased in mammographically dense breasts, where the sensitivity is reported as low as 30 to 48% [17], and the odds of developing breast cancer 17.8 times higher [18]. 

Hand held ultrasound (HHUS) has been used to optimize the detection of cancers in mammographically dense breasts but is limited due to technical factors, such as breast size, considerable user variability and reproducibility, technical skill, and time constraints, precluding HHUS as an effective screening modality for breast cancer [19–21]. Kelly described the use of volumetric breast ultrasound (VBUS) as an adjunct to mammography in the evaluation of nonpalpable breast cancers in asymptomatic women. VBUS with mammography resulted in an increase in diagnostic yield from 3.6 per 1,000 with mammography alone, to 7.2 per 1,000 by adding VBUS, resulting in a mammography miss rate of 3.6 per 1,000 [21]. However, one of the limitations of the study was that it did not isolate dense breasts as an independent risk factor for developing breast cancer, where the detection rate should be expected to be higher. VBUS is FDA approved in the United States for screening of women with dense breast parenchyma [22]. 

The purpose of this study was to demonstrate that VBUS increases the detection of nonpalpable breast cancers in mammographically dense breasts when used as an adjunct diagnostic modality in asymptomatic women. This resulted in the subsequent detection of cancers missed by mammography of smaller size and stage, justifying the basis for the judicious use of implementing VBUS in conjunction with mammography in the dense breast screening population. The tabulated data was extrapolated based on known mammography screening utilization to show a cost-benefit of additional VBUS as a population-based screening method. 

## 2. Methods

### 2.1. Selection of Participants

This study and the use of patient electronic health records were approved by an ethics committee appointed by the institute Board of Directors. The study design included two study groups, the control and test groups, in successive years. Each group was followed prospectively for 1 year. The control group consisted of women screened by digital mammography alone and stratified for breast density based on a Wolf classification of 50% or greater breast density (defined as the “mammographically dense breast” for the purpose of this study). The second group consisted of women initially screening by digital mammography as having mammographically dense breasts, followed by volumetric breast ultrasound (VBUS). Each group was carefully selected on the basis of breast density and having no major preexisting predictors of breast cancer, such as personal or family history of breast cancer, or BRCA gene positive. In addition, the test group patients were not included in the screening group so as to eliminate impact on the results of the test group patients. 

The control group, consisting of 4076 asymptomatic women designated as Wolf classification of 50% or greater breast density, underwent stand-alone screening digital mammography between January 2009 and December 2009 using digital mammography (Selenia, Hologic Inc., Bedford, MA USA). The sensitivity, specificity, positive predictive value, and negative predictive value for biopsy recommendation were determined, in addition to data collection regarding the size and stage of cancers missed by mammography.

The test group, consisting of 3418 asymptomatic women designated as Wolf classification of 50% or greater breast density, underwent stand-alone screening digital mammography between January 2010 and May 2011 using digital mammography (Selenia, Hologic Inc., Bedford, MA USA). This was followed by volumetric breast ultrasound (Somo-V, U-Systems, Sunnyvale, CA USA). The mammography-alone results were not used as control results in order to eliminate potential bias introduced by VBUS results on the mammography interpretations. In addition, mammography results were interpreted independently from VBUS results so as not to introduce bias. The sensitivity, specificity, positive predictive value, and negative predictive value for biopsy recommendation were determined, in addition to derived statistical data regarding the relative risk, and odds ratio for developing breast cancer.

### 2.2. Assessment of Mammographic Density

Mammographic density was assessed independently by radiologists on a dedicated mammography viewing workstation equipped with 5-Megapixel resolution. The radiologists were FDA qualified in mammography, with at least 10 years experience in breast ultrasound, 24 months of which included VBUS. Two radiologists interpreted both the mammography and VBUS examinations under identical viewing conditions of 5-Megapixel resolution. The mammograms and VBUS studies were double read by two radiologists, with final consensus determination for each case. Mammograms were evaluated according to one of five categories of density (0%, 1 to 24%, 25 to 49%, 50 to 74%, and 75 to 100%) and only mammograms with breast density of 50% or greater were included in the control and test study groups.

### 2.3. Volumetric Breast Ultrasound Evaluation

Volumetric breast ultrasound (VBUS) is a computer-based system for evaluating the whole breast. The whole breast ultrasound system (Somo-V, U-Systems, Sunnyvale, CA USA) was used in combination with a 6 to 14 MHz broadband mechanical transducer attached to a rigid compression plate and arm, producing over 300 images per image acquisition obtained as coronal sweeps from the skin to the chest wall. The mechanical arm controls transducer speed and position, while a trained ultrasound technologist maintains appropriate contact pressure and vertical orientation to the skin. Interpretation and reporting time for an experienced radiologist is approximately 10 minutes per examination. The radiologist has cine functionality to simultaneously view breast images in the coronal, sagittal, and axial imaging planes.

### 2.4. Data Collection

VBUS scan data was collected for location and size of breast masses and recorded in a radial or clock orientation consistent with American College of Radiology reporting lexicon. Studies were reported according to the American College of Radiology Breast Imaging Reporting and Data System (BI-RADS) six-point scale (0 = incomplete, needs additional assessment; 1 = normal; 2 = benign; 3 = probably benign; 4 = suspicious; 5 = highly suggestive of malignancy) [23, 24].

For BI-RADS scores of 1, 2, and 3 on ABUS, patients were followed prospectively for 1 year to exclude cancers missed on both mammography and VBUS. For BI-RADS scores of 4 and 5, stereotactic hand held ultrasound (HHUS) biopsy was performed using 14 gauge or larger percutaneous biopsy. HHUS was employed because VBUS is presently not equipped with biopsy capability. If a benign nonhigh risk lesion was diagnosed, such as simple breast cysts, no further tissue sampling was performed. All noncystic lesions were biopsied. Cystic lesions were identified as anechoic, thin walled lesions with posterior acoustic enhancement. All pathology proven breast malignancies were further staged using contrast volumetric/whole breast MR imaging (1.5T HDe version 15.0/M4 with VIBRANT software, GE Medical Systems, Waukesha, WI, USA.) with computer-assisted detection (CADStream software, Merge Healthcare, Belleview, WA, USA). A final pathological stage was assigned by the pathologists in the usual manner in accordance with the American Joint Committee on Cancer (AJCC) TNM system guidelines. The pathologists were blinded to patient participation in the study and the method of cancer detection.

### 2.5. Statistical Analysis

Calculations were made of the sensitivity, specificity, positive predictive value (PPV), negative predictive value (NPV), relative risk, odds risk, and attributable risk of breast cancer using MedCal version 12.2.1 software. Exact 95% confidence intervals (CI) were calculated for diagnostic yield. Statistical methods involved the chi-square test statistic, which was used to compare the number of cancers detected by VBUS, based on the size of cancer. *P* values of less than 0.05 were considered to indicate statistical significance. Attributable risk (AR) was calculated according to the following formula: AR = (RR − 1)Pc/RR  , where RR denotes relative risk of greater than 50%, and Pc prevalence of density of greater than 50% in case patients [25–27].

## 3. Results

Comparable interobserver diagnostic reliability (Kappa value of 0.98) was observed with mammography and VBUS examinations. In the control group (*N* = 4076), the median age of participants with breast cancer (*N* = 19) at the time of biopsy was 54 years, distributed as follows: 26% (5 out of 19) cancers occurred in women younger than age 50, 63% (12 out of 19) in women 50 to 69 years, and 11% (2 out of 19) over the age of 70 years. All cancers (*N* = 19) were biopsy proven invasive ductal carcinoma.

The sensitivity and specificity of stand-alone digital mammography were 76.00% (95% CI: 54.87%–90.58%) and 98.2% (95% CI: 97.76%–98.59%). The positive predictive value was 20.43% (95% CI: 12.78%–30.05%) with a breast cancer prevalence rate of 0.60% (95% CI: 12.78%–30.05%). The cancer detection rate was 4.6 per 1,000, with mean tumor size detected by mammography (*N* = 19) of 21.3 mm. The average size of missed breast cancer (*N* = 6) was 22.3 mm. The node positivity rate was 5% (1 of 19 cases).

In the VBUS study group (*N* = 3418), the median age of participants with breast cancer (*N* = 42) at the time of biopsy was 57 years, distributed as follows: 17% (7 out of 42) cancers occurred in women younger than age 50, 64% (27 out of 42) in women 50 to 69 years, and 19% (8 out of 42) over the age of 70 years. 

The sensitivity and specificity of VBUS were 97.67% (95% CI: 87.67%–99.61%) and 99.70%, (95% CI: 99.46%–99.86%), respectively, in mammographically dense breasts. The positive predictive value of VBUS was 80.77% (95% CI: 67.46%–90.36%), with a breast cancer prevalence rate of 1.25% (95% CI: 0.91%–1.69%). The odds ratio of breast cancer in mammographically dense breasts determined by VBUS was 2.65 (95% CI: 1.54–4.57; *P* = 0.0004). The cancer detection rate was 12.3 per 1,000. A 2.6-fold increase in cancer detection rate was observed between ABUS added to digital screening mammography compared to stand-alone digital screening mammography. 

Invasive breast cancer accounted for 81% (42 out of 52) solid breast masses detected by VBUS, of which 93% (39 out of 42) were invasive ductal carcinomas, and 7% (3 out of 42) were invasive lobular carcinomas. The mean tumor size detected by VBUS in patients with breast cancer (*N* = 42) was 14.3 mm, distributed as follows: Stage 1A disease accounted for 83% (35 out of 42) of cases; 12% were stage 2A (5 out of 42), and 5% were Stage 3A (2 out of 42). Stage 3A disease was associated with multifocal disease in both cases, one of which also was level 1 axillary lymph node positive. The node positivity rate was 2% (1 in 42) of cases. The false positive rate of VBUS was 19.3%, with a negative predictive value of 99.97% (95% CI: 99.83%–100.00%). The pathologies associated with false positive results (*N* = 10) were fibroadenomas and atypical epithelial neoplasms. 

We also used our data to extrapolate the theoretical cost-benefit of VBUS screening applied to a large screening population in the United States. Our analysis relied on the following assumptions: (1) Global Centers for Medicare and Medicaid reimbursement rate of breast ultrasound of $71 [28]; (2) estimated mean doubling time of a missed cancer of 250 days at the 95th percentile [29, 30]. According to previously cited cancer kinetics models, a missed breast cancer should be clinically evident within 9 months [31]. When we considered the mean breast cancer size in our positive test subject group, 14.3 mm (*N* = 42), we extrapolated a theoretical missed cancer size of 29.2 mm at 9 months in mammographically dense breasts, representative of Stage 2 or greater disease. In control subjects, a mean breast cancer size of 22.3 mm was consistent with stage 2 breast cancer. Incremental treatment cost assumptions, based on the Global Centers for Medicare and Medicaid reimbursement rate between Stage 1 and Stage 2 breast cancer, were $24,002 and $34,469, respectively, for a cost differential of $10,467 [32]. Accordingly, the aggregate costs of screening 3418 VBUS patients in this study were $239,260, compared to the estimated aggregate costs of additional treatment in 26 potentially missed cancers (based on previously noted theoretical assumptions) of $275,557 based on a cancer miss rate of 0.77% (or 7.7 per 1,000). 

## 4. Discussion

Magnetic resonance imaging (MRI) has recently been recommended by the American Cancer Society (ACS) to screen women at very high risk of breast cancer [33]. Though highly sensitive, MRI is costly and does have some drawbacks, such as the risks from the required contrast media. MRI for breast cancer screening has also been characterized by lower specificity, as compared to mammography, with a higher rate of false positives, leading to further follow-up MRI and/or imaging-guided biopsy costs. For example, a study by Leach et al. reported MRI specificity of 81 percent, compared to 93 percent specificity in mammography [34]. Griebsch et al. reported MRI as having almost four times more recalls than mammography for women with high familial breast cancer risk, and 70 percent of the recalls did not involve cancer [35]. Because of lower specificity and higher cost, compared to mammography, MRI may not be optimal for breast cancer screening.

In our experience, volumetric breast ultrasound (VBUS) can be helpful, particularly in identifying small tumors, due to a 3 mm slice thickness and high pixel resolution on postprocessed volumetric scans. Characteristic features of malignancy on VBUS include a hypoechoic breast mass with irregular margins, “taller-than-wide” appearance, and acoustic shadowing. In these cases, MR imaging can provide a specific imaging application over VBUS in differentiating between invasive ductal carcinoma (IDC) from invasive lobular carcinoma (ILC) based on the enhancement characteristics of the tumor and signal properties on T2-weighted images. MR imaging shows characteristic desmoplasia as signal loss on T2-weighted images (the “thumbprint sign”) which can be a reliable indicator that the tumor is IDC rather than ILC [36].

Following are the clinical criteria for ABUS screening, which are the clinical indications for ordering a VBUS examination:

- as a supplement to mammography, screening for occult cancers in certain populations of women (such as those with dense fibroglandular breasts and/or with elevated risk of breast cancer);
- imaging evaluation of nonpalpable masses in women under 30 years of age who are not at high risk for development of breast cancer, and in lactating and pregnant women;
- BI-RADS (American College of Radiology Breast Imaging Reporting and Data System) scoring classification class III, heterogeneously dense, with 50% to 74% or 75% to 100% breast density on mammography, without palpable mass.

**Figure 1:**
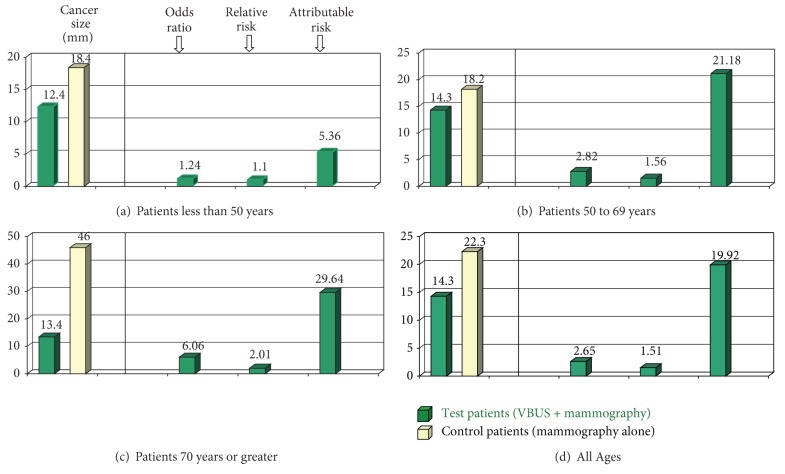
Breast cancer staging and risk assessment by screening method detection. Box plots comparing case patients and control subjects according to age, (a) through (d). Tumor sizes are shown as a function of the odds ratio, relative risk, and attributable risk for each age category. Bars represent the highest and lowest observed values with respect to individual variables (individually labeled with arrows).

Table 1 shows the distribution of breast cancer size according to age in the control and test study groups. The test group showed no statistical difference between size of the cancer and patient age at presentation. A significant increase in tumor size in the over 70 patients in control subjects was attributed to the more advanced tumor stage at presentation.  Table 2 shows that stand-alone digital mammography was less sensitive than VBUS in breast cancer detection, with a 4-fold increase in positive predictive value of VBUS compared to stand-alone mammography in dense breasts. Our results showed that mammographic density of 50% or more was associated with an increased risk of breast cancer and resulted in a significant miss rate in asymptomatic women.  Table 3 shows a statistically significant age-related attributable risk of developing breast cancer for mammographic density of 50% or greater. These observations are consistent with other studies which have shown an increased risk of breast cancer in dense breasts following negative mammography screening [2, 3, 17, 18]. We observed that breast cancer risk was highest in patients over age 70, where increased breast density was associated with an attributable risk of 29.6 (95% CI: 21.5–40.8).  Figure 1 shows box plots comparing case patients and control subjects according to age, with tumor sizes shown as a function of the odds ratio, relative risk, and attributable risk for each age category.

Our study also showed that volumetric breast ultrasound (VBUS) was an effective screening modality in mammographically dense breasts. Our extrapolated data suggest a breast cancer miss rate of 7.7 per 1,000 in mammographically dense breasts in asymptomatic women, which is higher compared to the cancer miss rate of 3.6 per 1,000 reported by Kelly et al. using VBUS [21]. We attribute the increased breast cancer miss rate due to breast density, which was isolated as the principal risk factor in our study. Other studies have shown that the attributable risk of breast cancer for a mammographic density of 50% or greater was 40% for all cancers detected less than 12 months after a negative screening mammogram, and as high as 50% in women less than the age of 50. This marked increase in the risk of breast cancer associated with mammographic density of 50% or greater up to 12 months following screening directly reflects cancers that were present at the time of screening but went undetected due to masking by dense breast parenchyma [37–41].

In the final analysis, there is the issue of the theoretical cost-benefit of adding VBUS screening to mammography in an otherwise healthy population. The importance of screening mammographically dense breasts with VBUS has particular relevance based on the small size and early stage of breast cancers. Our study showed a mean tumor size of 14.3 mm, representing stage 1 disease, which was present in 81% of patients. From our data, we derived theoretical population-based costs as a basis for the cost-benefit of VBUS in the United States population. Our study compared the incremental costs of screening versus the costs of added treatment related to a change in the staging of missed cancers from Stage 1 to Stage 2. 

The costs of additional treatment outweighed the costs of screening by $32,808, which calculated to $9.60 added healthcare cost per patient in the 3418 participants in the study. In the United States, 48 million mammograms were performed annually, with a reported estimated miss rate of 10% [42]. When comparing control versus test patients, our study suggests a theoretical miss rate of 7.7 cancers per 1,000 mammograms, or 0.77%, which is considerably lower than the reported missed rate of 10%. Based on these theoretical assumptions, annual added VBUS screening of the entire USA population would cost $3.40 billion. However, in actual practice, VBUS would be used only in the mammographically dense breast, which would potentially reduce the screening costs by at least a factor of 0.8, bringing the cost closer to $2.72 billion. By contrast, the incremental costs of added treatment associated with stage 2 compared to stage 1 breast cancer in the USA population would be $3.82 billion, assuming a conservative cost basis of $10,467 per patient. The cost-benefit of early detection of stage 1 disease results in a theoretical per capital annual cost savings of $22.75 per screened patient in the USA population, according to our model. However, we have no actual or derived data to support improved breast cancer mortality with the addition of VBUS as a universal screening modality. This is one of the major limitations of our study because actuarial analyses used to justify screening modalities are typically based on mortality statistics. With respect to five-year survival statistics between stage 1 and stage 2 breast cancers, of 98% and 80%, respectively, one could construe the potential for a theoretical quality-of-life benefit based on judicious VBUS screening. Another limitation of our study is the relatively small screening population used in our study, emphasizing the need for continued research in order to validate VBUS as a viable and cost-effective population-based screening modality, which should be stratified for other risk factors for breast cancer, such as personal or family history of breast cancer, BRCA genetic results, environmental factors (late parity, previous exposure to ionizing radiation, exogenous estrogen, smoking, and alcohol use), early menarche/late menopause, and ethnic/racial differences.

At most imaging centers, mammography is the only screening method for breast cancer detection. Our study corroborates with the data derived from other studies that the principal mechanism for breast cancer in dense breast parenchyma is not rapid growth, but rather, the masking of coincident cancers that are missed on screening mammograms. These findings further suggest that the addition of mammographic screening in patients with dense breast parenchyma is likely not to increase diagnostic yield in the detection of breast cancers. Therefore, emphasis should be placed on alternative imaging techniques for such women. To conclude, our study of a small representative dense breast screening population showed that the addition of VBUS was more effective than digital mammography alone. This study provides a platform for using VBUS as cost-effective approach to breast cancer detection in the judicious screening of asymptomatic women with excessive mammographic density, in whom the greatest risk is between screening mammography examinations.

## Figures and Tables

**Table 1:** Breast cancer size according to method detection.

Age	Control subjects (mammography alone) (*N* = 19)	Average tumor size (in mm)	Test subjects (mammography with VBUS) (*N* = 42)	Average tumor size (in mm)
<50 yr	5	18.4	7	12.4
0–69 yr	12	18.2	27	14.3
≥70 yr	2	46.0	8	13.4

Totals	19	22.3	42	14.3

**Table 2:** Detection of breast cancer according to method.

	Control subjects mammography alone	Test subjects mammography + VBUS
	*N* = 4076; 95% CI	*N* = 3418; 95% CI

Sensitivity	76.00% (54.87%–90.57%)	97.67% (87.67%–99.61%)
Specificity	98.21% (97.76%–98.59%)	99.70% (99.46%–99.86%)
Positive likelihood ratio	42.43 (30.95–58.16)	330.73 (177.80–615.17)
Negative likelihood ratio	0.24 (0.12–0.49)	0.02 (0.00–0.16)
Disease prevalence	0.60% (0.39%–0.89%)	1.25% (0.91–1.69%)
Positive predictive value	20.43% (12.78%–30.05%)	80.77% (67.46%–90.36%)
Negative predictive value	99.85% (99.68%–99.95%)	99.97% (99.83%–100.00%)

**Table 3:** Risk of breast cancer according to method detection.

Age (years)	Negative outcomes		Positive outcomes		Odds ratio (95% CI)	Relative risk (95% CI)	Attributable risk (95% CI)
	Test (*N* = 3376)	Control (*N* = 4057)	Test (*N* = 42)	Control (*N* = 19)			
<50	776	688	7	5	1.24 (0.39–3.92)	1.10 (0.68–1.77)	5.36 (3.31–8.62)
50–69	2198	2759	27	12	2.82 (1.42–5.58)	1.56 (1.26–1.92)	21.18 (17.10–26.06)
≥70	402	610	8	2	6.06 (1.28–28.72)	2.01 (1.46–2.77)	29.64 (21.52–40.84)

Totals	3376	4057	42	19	2.65 (1.54–4.57)	1.51 (1.27–1.79)	19.92 (16.75–23.61)
